# Protein Microarrays and Biomarkers of Infectious Disease

**DOI:** 10.3390/ijms11125165

**Published:** 2010-12-16

**Authors:** Mohan Natesan, Robert G. Ulrich

**Affiliations:** Department of Immunology, United States Army Medical Research Institute of Infectious Diseases, Frederick, MD 21702, USA; E-Mail: rulrich@bioanalysis.org

**Keywords:** protein microarray, infectious diseases, biomarkers

## Abstract

Protein microarrays are powerful tools that are widely used in systems biology research. For infectious diseases, proteome microarrays assembled from proteins of pathogens will play an increasingly important role in discovery of diagnostic markers, vaccines, and therapeutics. Distinct formats of protein microarrays have been developed for different applications, including abundance-based and function-based methods. Depending on the application, design issues should be considered, such as the need for multiplexing and label or label free detection methods. New developments, challenges, and future demands in infectious disease research will impact the application of protein microarrays for discovery and validation of biomarkers.

## 1. Introduction

**Figure 1.**
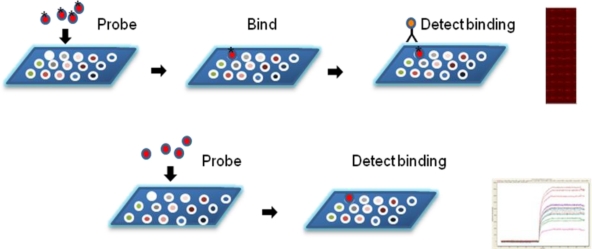
**Measuring probe interactions with microarrayed proteins**. Top: Detecting binding events using a fluorescently-labeled secondary probe, such as an antibody, and the resulting laser-scanned image. Bottom: Label-free interactions detected by surface plasmon resonance, resulting in a sensorgram of binding kinetics.

Large-scale genome sequencing projects first advanced knowledge of the theoretical composition of proteomes and led to the development of DNA microarrays for studying gene transcription at the organism-scale. Using genome sequence data to guide the direct examination of proteomes then enabled the study of host-pathogen interactions occurring beyond the level of gene transcription. It soon became evident that direct correlations between gene expression and protein abundance were rare [1,2], driving the development of new approaches to study complex proteomes. Protein microarrays are ideally suited to serve this purpose and have enormous potential applications in biomarker discovery, diagnosis, vaccine development, and drug discovery for infectious diseases. Fluorescence-based detection of protein interactions is similar to gene array methods, and data analysis often employs approaches previously developed for genome and transcription studies. The number of proteins that can be printed on a single microarray surface also approaches the same upper limits as nucleic-acid based systems. Multiplexing of fluorescent probes is limited by the ability to separate signals of overlapping emission spectra. Generally, pair-wise comparisons of 2–3 differentially labeled, experimental and control samples can be analyzed. For example, serum IgG and IgM binding to arrayed proteins can be independently probed by fluorescently labeled secondary antibodies, and detected by a confocal laser scanner (such as Genepix, Molecular Devices, Sunnyvale, CA, USA). A complete high-throughput screening of thousands of interactions can be performed accurately and rapidly (Figure 1). Direct labeling of probes may also be used, though structure and function may be adversely affected. Most label-free techniques [3] provide real-time measurements and in some cases yield kinetics (Figure 1), providing further insight into molecular interactions. Surface plasmon resonance (SPR) [4], nanowire surfaces [5], and mass spectrometry [6] are examples of label-free methods that are applied in the field of protein microarrays. Of these, only a limited number of SPR-based instruments are currently available for analysis of complex protein microarrays. SPR imaging (SPRi) is a more recent and promising development [3,7]. While planar arrays that use slides or chips for immobilizing capture molecules are most common, suspension arrays based on microbeads have many important applications. The xMAP technology developed by Luminex Corporation (Austin, TX, USA) uses 5.6 micrometer polymer beads infused with two fluorescent dyes at different ratios to yield up to 100 distinct bead sets. The binding measurements are performed by flow cytometry with two lasers, one for the identification of the bead and the other for the sample. Bead-based arrays are very useful for standardizing assays, with an upper limit of 80 independent immobilized probes.

Protein microarrays are increasingly used in infectious disease research to identify new biomarkers that are involved in the disease process or that are targets of immune responses [8–13]. In contrast with other proteomic techniques such as 2-D gel electrophoresis and mass spectrometry, the arrayed probes are known, conforming easily to high-throughput applications that require examination of the entire proteome of an infectious agent. A variety of formats have been developed for the study of pathogen proteomes. Capture and reverse-phase are examples of abundance-based microarrays produced by spotting either antibodies [10] or antigens [14] on a substrate. The arrays can be probed with serum, plasma, and other biological samples to detect binding interactions. Microarrays based on well-characterized antibodies are specifically useful for identifying biomarkers and quantification of proteins. Aptamers [15], affibodies [16], or engineered antibody fragments [17] are alternative capture molecules. Antigen arrays find applications in serodiagnosis of disease, antibody-response profiling, and evaluation of immunity after vaccination. In reverse-phase protein microarrays (RPPMs), small amounts of biological samples from cells, tissues, sera, or plasmas are directly printed on the slide [18,19]. These arrays are probed with a single protein of interest that is detected by labeled antibodies. RPPMs were successfully used for the screening of cell-signaling pathways and posttranslational modifications of proteins [20–22]. The biochemical activities of arrayed proteins have also been analyzed [23] by enzyme activity or substrate activity, and protein interaction with other proteins, nucleic acids, or drugs [24–26].

## 2. Proteome Microarrays and Host Antibody Responses

**Figure 2.**
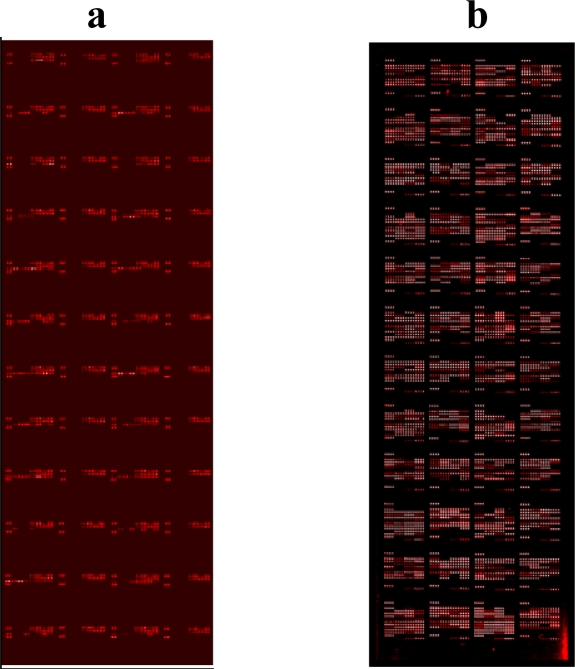
**Pathogen proteome microarrays**. Confocal laser scanner image of proteins spotted in duplicate onto microarray slides, visualized using a rabbit anti-GST antibody bound to Cy5-labeled anti-rabbit antibody. (**a**) Vaccinia virus; (**b**) *Yersinia pestis.*

The host antibody response provides a signature of pathogen proteins displayed by infectious diseases that can be captured and detected by microarrays. Our studies with vaccinia virus [12] and the bacterium *Yersinia pestis* [11] exemplify the application of antigen microarrays for the analysis of immune responses (Figure 2). Vaccinia is a large DNA virus (Orthopoxviridae) that replicates in the cytoplasm of host cells from genomes encoding 150–300 proteins. The virion is comprised of approximately 100 proteins. Most phenotypic variability occurs in proteins encoded in the terminal regions of the genome that are associated with host virulence or immune evasion. Some of these terminal-region proteins are secreted during cell infection and interfere with host immunity by binding complement factors, cytokines, and chemokines, while others interfere with signaling pathways regulating host gene expression and apoptosis. To construct a proteome microarray, genomic DNA of the Copenhagen (NC_001559.1) vaccine strain (Dryvax) was used as the template for PCR amplification of the 273 open reading frames (ORF). Gateway recombination cloning (Invitrogen, Carlsbad, CA, USA) was employed to facilitate high-throughput production of proteins from all ORF clones. All DNA clones were sequence-verified through the entire length of their inserts. Baculovirus-based expression was used to produce the recombinant viral proteins as GST-tagged fusions to ensure high yield of properly folded proteins with posttranslational modifications that are similar to those encountered in the human host. The GST-tagged proteins from cell lysates were affinity purified to 90% homogeneity in a single step by using glutathione-agarose in 96-well plates and analyzed for correct size and abundance by Western blots. Virus and control proteins were printed onto glass slides coated with a thin layer of nitrocellulose. Ultimately, 95% of the proteome was successfully expressed, purified, and arrayed.

A standard assay for measuring antibody interactions with proteins of the vaccinia proteome microarray was first developed with a pool of therapeutic human sera collected from vaccinia-immune individuals (VIg) and this data was compared to results obtained from individuals vaccinated against smallpox using Dryvax. The assay consisted of incubating a dilution of serum on the microarray surface, washes, and finally detection with a fluorescently labeled anti-Ig antibody. Fluorescent images were captured by scanning with a confocal laser (GenePix 4000B; Molecular Devices). Because only a very small sample volume (1–2 μL) is required, this approach is ideal for limiting the amount of sample consumed, often necessary with clinical material. A high level of reproducibility with a very low background was apparent in repetitive assays that confirmed previously reported antigens and identified new proteins that may be important for neutralizing viral infection. Incubation of the microarray with VIg identified nine proteins that consistently bound antibody, while antibody interactions with all other proteins were insignificant, requiring no further treatment to suppress non-specific signals. These antigens were also recognized by antibodies from individual subjects following primary smallpox vaccination and were diverse in function, consisting of regulatory, surface, core, and secreted proteins. The identical vaccinia proteins clustered into proteins recognized by primary and secondary vaccinated subjects based solely on signal intensities. The vaccinia proteins C3L and I1L were not previously reported as antibody-recognized antigens. The nine antigenic proteins we identified did not bind antibody from non-vaccinated sera, confirming the specificity of these antibody-antigen interactions. However, O2L and H7R were reactive with antibodies from both VIg and non-vaccinated control sera, suggesting that these were cross-reactive or non-specific interactions. These results indicated that only a small subset of proteins present within the complex orthopoxvirus proteome was associated with antibody responses. In addition, the human response to individual proteins was variable as sera from more than half of the subjects contained IgG that recognized >4 vaccinia proteins, while the remaining samples recognized one to three proteins. Further, antibodies from most individuals recognized a greater number of viral proteins after a boost vaccination compared to a single vaccination, suggesting that repeated infection expands the total number of proteins recognized by IgG. A previous report [27] similar to our study on vaccinia [12], analyzed the serological response to vaccinia and smallpox, observing that a significant amount of the antibody response to vaccinia was not involved in the virus neutralization, but could be used as potential markers for diagnostic and vaccine development. A later report [28] evaluated an attenuated smallpox modified vaccinia virus Ankara (MVA) an alternative to Dryvax vaccine by comparing antibody profiles in the sera of humans and monkeys. The overall immunogenicity of MVA was reported to be comparable to that of the Dryvax vaccine and the study concluded that MVA may be a useful alternative to Dryvax.

Our vaccinia virus microarray was expanded to allow analysis of additional poxviruses. Monkeypox is a zoonotic viral disease that occurs primarily in Central and West Africa. Immunity to monkeypox is provided by vaccination against smallpox caused by the closely related variola virus. To differentiate antibody responses to monkeypox virus infection from human smallpox vaccination, we developed a protein microarray covering 92–95% (166–192 proteins) of representative proteomes from monkeypox viral clades of Central and West Africa, including 92% coverage (250 proteins) of the vaccinia virus proteome as a reference orthopox vaccine. Serum IgG of cynomolgus macaques that recovered from monkeypox recognized at least 23 separate proteins within the orthopox meta-proteome, while only 14 of these proteins were recognized by IgG from vaccinated humans. There were 12 of 14 antigens detected by sera of human vaccinees that were also recognized by IgG from convalescent macaques. The greatest level of IgG binding for macaques occurred with the structural proteins F13L and A33R, and the membrane scaffold protein D13L. Significant IgM responses directed towards B16R, F13L, and A33R of monkeypox virus were detected before onset of clinical symptoms in macaques. Results from this study suggested that antibodies from vaccination recognized a small number of proteins shared with pathogenic viral strains, while recovery from infection also involved humoral immunity to antigens uniquely recognized within the monkeypox virus proteome (unpublished data).

To identify antibody biomarkers that could distinguish plague from infections caused by other bacterial pathogens [11], we developed a microarray comprised of the proteome from the bacterium *Yersinia pestis*. The chromosome of *Y. pestis* CO92 encodes approximately 3885 proteins, while an additional 181 are episomally expressed by pCD1, pMT1, and pPCP1. For comparison, the proteome of *Y. pestis* KIM contains 4202 individual proteins, 87% in common with CO92, and the closely related enteric pathogen *Y. pseudotuberculosis* contains approximately 4038 proteins (chromosome plus plasmids). The microarray was comprised of proteins representative of over 75% of the 4066 ORFs present within the *Y. pestis* genome. In a manner similar to the vaccinia microarray, the *Y. pestis* proteins were produced as full-length polypeptides fused to GST as an affinity isolation tag. An *in vitro* translation method, based on *E. coli* lysates, was used to express the proteins in a gram-negative background. The ORF clones were fully sequenced to confirm quality and identity. The affinity-purified proteins were characterized by SDS gels and Western blots probed with an anti-GST antibody. Different approaches for studying the antibody repertoire for plague in rabbits and non-human primates were investigated. Based on results from experiments using the *Y. pestis* proteome microarray, we identified new candidates for antibody biomarkers of bacterial infections and patterns of cross-reactivity with a panel of other gram-negative pathogens [11]. We were able to cluster the proteins recognized by the antibodies into three groups: common proteins of all gram-negative bacteria, combinations of proteins unique to one pathogen, and proteins unique to one pathogen. This example demonstrates the power of a high-density proteome microarray to dissect the complex immunological relationships among a group of related human pathogens. Convalescence sera from vaccinated non-human primates that survived an otherwise lethal aerosol challenge with *Y. pestis* CO92 (plague) or *Bacillus anthracis* Ames spores (anthrax) were also examined. In addition to the CaF1 and LcrV proteins comprising the vaccine components, a subset of proteins from the *Y. pestis* proteome were recognized by antibodies from plague survivors and none of these were detected with antibodies from anthrax survivors or non-challenged controls. Signature patterns of antibody recognition were identified that reflected the orthologous relationships among proteomes of these bacteria. A high degree of cross-reactivity in the antibody response to *Burkholderia* and related bacteria was also previously reported [29]. Further, a protein microarray consisting of 4% of *Y. pestis* proteins was used by Li *et al.* to profile antibody responses to live plague vaccine in rabbits [30], sentinel animals [31], and plague patients [32]. Predominant responses were observed for 11 proteins in addition to F1 and V antigens in rabbits. In plague patients, they identified 14 proteins that were potential serodiagnostic biomarker candidates and found antibody responses from different plague foci responsible for different clinical symptoms.

Additional microarray studies of antibody responses to bacterial pathogens were reported. Sundaresh *et al.* [33] developed a protein microarray with 244 of the most reactive *Francisella tularensis* proteins derived from a larger chip. In this study, they identified a set of immunodominant antigens from *F. tularensis* patients suitable for diagnosis development. In another study [34], a similar protein microarray approach was employed to find immunodominant antigens in mice vaccinated with killed *F. tularensis* vaccine and challenged with a virulent *F. tularensis* strain. The study found 31 antigens in addition to 12 known immunodominant antigens of *F. tularensis* discovered by other methods. This was also the first published report to use protein microarrays for studying protective immune responses to an infectious agent. Immunoreactive antigens of *Coxiella burnetti,* the causative agent of Q fever, were identified using a protein microarray consisting of 75% of the *C. burnetti* proteome assembled by using a cell free protein expression system [35]. Serum from Q fever patients and individuals vaccinated with Q-Vax vaccine strongly reacted to 50 antigens of *C. burnetti* that were confirmed by ELISA including antigens isolated from an *E. coli* expression system. In yet another study, human and goat antibody responses to *Brucella melitensis* infection were analyzed [36], finding that there was differential recognition of antigens by the two host species. The serodiagnostic proteins were of potential value for diagnostic purposes as brucellosis is diagnosed by measuring the antibodies to lipopolysaccharide (LPS), which cannot distinguish between past and present infection in endemic areas. Antibody response to Lyme borreliosis disease in humans and mice was studied by Barbour *et al.* [37], using a protein microarray comprising 80% of the proteins from *Borrelia burgdorferi*. A comprehensive analysis of the sera from *B. burgdorferi* patients and rodents revealed that only a small set of antigens elicited antibody responses in both hosts, while the majority of *B. burgdorferi* proteins did not induce antibodies. It is also possible to focus on specific subsets of antigens identified by bioinformatics. For example, a protein array produced from the outer membrane proteins of *Pseudomonas aeruginosa* was constructed [38] to study the immune response in patients, and several antibody-binding antigens were identified by this group as potential diagnostic markers. In another example, a recombinant *Niesseria meningitidis* protein array was used for screening serum from meningitis patients [39] for immune responses to phase-variable expressed proteins.

## 3. Protein-Protein Interactions (Non-Antibody)

The development of new proteomics methods has been driven in part by the need to identify new types of infection biomarkers. Though studies of antibody responses dominate the field, protein microarrays should be considered as an alternative approach to examine other catagories of host-pathogen interactions. Research areas with well-established cell proteomics data are prime targets. For example, because many of the host factors associated with phagosomes have been identified [40], protein microarrays may provide a convenient model system to examine interactions with pathogen proteomes associated with this portal of entry. In a similar manner, targeted microarrays comprised of pathogen proteins may be used to examine host interactions. The few published examples from this developing field of study should be mentioned. In one report, RPPM along with other techniques [41] were used to examine phosphoprotein signaling pathway in primary human small airway epithelial cells infected with *Bacillus anthracis*, identifying reduced AKT (protein kinase B) phosphorylation as a contributor to increased bacterial survival and pathogenicity. In another study [42], proteins from Group A and B streptococci were arrayed on a chip and probed with human fibronectin, fibrinogen, and C4 binding protein, all well-known targets of gram-positive bacteria.

## 4. Alternative Methods

The use of purified and well-characterized proteins to assemble the microarray will insure the highest quality and most reproducible results. However, the high level of quality control required to sequence, purify, and characterize elements of a proteome microarray in the examples we described may not be as important for provisional screening purposes. A coupled transcription/translation reaction was previously reported as a rapid method to develop protein microarrays against a number of infectious organisms [43,44], bypassing sequencing and purification steps. In this technique, both transcription and translation occur simultaneously to synthesize proteins in parallel from PCR products, directly on the chip. Presumably, results obtained by these methods will require follow-up studies with more rigorous controls for confirmation. The protein *in situ* array (PISA) developed by He and Taussig [45] first produces DNA constructs from a PCR of the protein of interest and a T7 promoter for translational initiation followed by immobilization using a N or C terminal tag sequence. Another approach [25,46] called nucleic acid programmable protein array (NAPPA) included slides coated with avidin to capture biotinylated plasmid DNA for synthesizing proteins. Proteins with fused tags were synthesized and immobilized on the chips coated with molecules to bind the tags [25]. He *et al.* [47] described a technique called DNA array to protein array (DAPA) where they utilized a single DNA array to make multiple copies of the same protein array. In one example, a self-assembled NAPPA-based on a cell-free expression system was used for constructing a protein microarray of all 69 proteins produced by varicella zoster virus (VZV), the cause of chickenpox [48]. Three sets of antigens in the VZV proteome were identified from human sera that may be useful in defining the clinical status of the infection and diagnostics. Another VZV protein array produced from an *E. coli* expression system was later used to detect antibodies in human sera reactive to VZV viral proteins [49]. The ORF68 (gE) antigen identified in the study showed high-confidence for determination of the serological response to VZV.

Although these alternative *in situ* synthesis methods are rapid, they are not without significant shortcomings. It is difficult to characterize proteins expressed on the chip in contrast to direct spotting of purified proteins. Two studies investigated the antibody response to VZV by protein arrays made from either NAPPA cell-free expression system [48] or *E. coli* expression system [49]. Antibodies from the sera of patients recognized three microarrayed VZV proteins produced recombinantly with *E. coli*, whereas these identical proteins were not recognized on a NAPPA protein expression-based protein microarray. Interestingly, these proteins were abundantly expressed in the NAPPA protein microarrays, suggesting that they did not fold correctly to expose their linear epitopes for binding. Despite these limitations, microarrays based on *in situ* synthesis offer simplicity, efficiency, and high-throughput capabilities.

The relatively small proteomes of most viruses increase in diversity by considering variations in clinical isolates. For example, while human papilloma virus produces only a few proteins, sequences for these may diverge in the more than 100 strains of the virus. A protein microarray developed with 13 human papilloma virus types [50] was used to examine patient sera compared to asymptomatic subjects. The study identified E7 proteins as the most reactive antigens in the patient group. However, the E7 proteins were not useful as a diagnostic biomarker because the differences between patients and asymptomatic subjects were not significant. In another report, a bead suspension multiplex array (Luminex xMAP) was used to study serological responses to 27 antigens of human papilloma virus [51]. The data correlated well with ELISA results and in addition the bead array was able to measure weak antibody responses. A protein array constructed from six coronaviruses including severe acute respiratory syndrome (SARS) was used for screening serum samples for virus-specific antibodies [52]. The microarray data predicted SARS infection in 90% of the patients correctly with a specificity of 93%.

## 5. Peptide and Protein Domain Microarrays

The high efficiency, low cost, and high-throughput nature of chemically synthesized peptides are significant advantages compared to production of recombinant polypeptides. Therefore, peptide microarrays have been explored as another alternative to recombinant proteins, with a limited degree of success. A chemically synthesized peptide microarray representing the major antigens of hepatitis B and C viruses, human immunodeficiency virus, Epstein-Barr virus, and syphilis was constructed on a glass slide and antibody responses were simultaneously detected [53]. The assay showed very high sensitivity and specificity for the diagnostic identification of these viruses. Another proof of principle study [54] was reported to identify diagnostically relevant peptides using a peptide array deduced from bioinformatics data for *Echinococcus granulosa* (tapeworm) infection, achieving only 57% sensitivity and 94% specificity compared to ELISA.

As an alternative to full-length polypeptides, expression and purification of protein domains may improve yield of stable products for use in microarrays. In one reported study, a total of 212 protein domains (SH3, SH2, PDZ *etc.*) were immobilized on nitrocellulose and screened with peptides [55]. The results proved that the immobilized domains were stable and could be used for binding studies. Kaushansky *et al.* [56] constructed an array based on protein interaction domains such as *Src* homology, phosphotyrosine binding domains, and mouse PDZ domains produced from recombinant *E. coli*. These arrays required low sample consumption and may reduce false positive rates. Advanced knowledge of the specific recognition events will be required to substitute peptides or single protein domains for full-length proteins to ensure that important epitopes are not missed for the purpose of studying antibody interactions.

## 6. Quality Control

Protein stability is an important and particularly challenging factor in using protein microarrays. The high-throughput nature of construction tends to sacrifice detailed knowledge about individual proteins. There are few methods available to assess folding and functionality of all arrayed proteins immediately before use. Recombinant proteins constructed with fusion tags to facilitate folding during translation offer a partial solution, especially if proper folding of the fusion protein can be readily assessed. For example, unfolded glutathione-S-transferase (GST) will not bind to glutathione, the tripeptide ligand of GST, hence purification schemes employing GST fusions will favor proteins with stable structures. Fusion tags also provide a convenient marker for monitoring quality control of protein spotting (Figure 2). However, the fusion tag may hinder protein accessibility or alter function in other ways. Because proteins may denature during printing and storage, enzymes or other denaturing-sensitive proteins that can be assessed after printing should be included in the microarray.

## 7. Antibody Microarrays

Antibodies are a natural choice to be used as capture molecules due to their specificity, affinity, ease of production, and potential for engineering. For probe development, the analysis of antibody specificity and cross-reactivity [57–59] can be simplified by screening against target or off-target proteins printed as microarrays. Antibody arrays are routinely used in the field of biomarker discovery and validation. The detection of binding molecules to an antibody array is usually done by direct labeling of proteins in the sample (serum, cell, or tissue) or by a sandwich format similar to ELISA. For example, protein abundance in cells was determined by Haab *et al.* [60], using 115 antigen and antibody reactions. This was the first published report to use a two-color differential labeling of samples in a protein microarray. The microarray format is also convenient for standardizing the routine analysis of analytes present in screening assays, such as cytokines released by cell cultures [61]. An antibody-based microarray for the multiplexed detection of cholera, diphtheria, staphylococcal enterotoxin B, tetanus toxins, anthrax protective antigen, and lethal factor was reported [62]. A competition assay between labeled and unlabeled toxins in serum was used to simultaneously detect all the toxins in the antibody microarray. In another report, a sandwich fluorescence immunoassay based on an antibody microarray format was developed for the capture and detection of *E. coli* O157.H7 [63], and later to screen large number of food samples in a high-throughput manner to simultaneously detect *E coli* O157:H7 and *Salmonella typhimurium* [64].

The major drawback of capture microarrays is that few well-defined and high-quality antibodies against microorganisms are available. Engineering antibody fragments is a potential alternate approach to accelerate the development of antigen-capture molecules. Antibody fragments such as the Fab portion of IgG [65], phage display libraries or recombinant single chain variable fragment (scFv) containing the antigen-binding motif can be used in place of antibodies in protein microarray development [66,67]. A decreased cross-reactivity was also observed with fragments as compared to full-length antibodies.

## 8. Microarray Printing

Thin-layered nitrocellulose or chemically modified 2-D surfaces (glass, silicon, gold, and polymer) are generally used for immobilization of microarrayed proteins. Buffer additives like glycerol, polyethylene glycol, or sugars that prevent drying of the proteins and choice of buffers should be carefully selected to match the type of surfaces used for arraying. Alternatively, gel-based 3-D surfaces made from polyacrylamide or agarose provide a hydrophilic environment [68]. Regardless of the printing substrate selected, microarrayed proteins must retain functionality and stability during preparation and use. Though it may not be possible to directly examine all elements of an array, it is important to include printing controls of interacting proteins that can be tested for functionality before use. There are several methods by which proteins are spotted onto substrates. The choice of technology used impacts spotting consistency, speed, spot diameter, and ease of use. In turn, the final spot quality required for printed proteins will depend on the method of detection. Control of the laboratory environment to maintain constant temperature, humidity, and clean-room conditions will vastly improve printing consistency. Printing with solid pins (for example Stealth Pin, Arrayit Corportion, Sunnyvale, CA, USA) relies on capillary forces to release spots on contact with the surface, resulting in 60–600 μm diameter spots depending on buffer composition and pin diameter. Dip-pen lithography (DPN) uses atomic force microscopy microcantilevers to deposit spots in the range of 1–60 μm diameter (Nano eNabler, Bioforce Nanosciences, Ames, IA, USA) and inkjet printers (for example Arrayjet, Roslin, Scotland) use piezoelectric elements to transfer the protein solution in the form of droplets to the target surface, resulting in spot diameters of 80–150 μm. Both pin and inkjet spotting methods deposit a very small amount of protein, requiring highly concentrated samples. Unfortunately, highly concentrated samples may fail to adsorb completely on the surface and tend to spread during hydration. A new continuous flow microspotting method (Wasatch Microfluidics, Taylorsville, UT, USA) uses microfluidic channel networks to continuously circulate protein samples over a spot to achieve uniform and maximum protein adsorption [69]. This technique may be especially useful for dilute and crude protein sample arraying.

## 9. Labeled Detection of Binding Events

Fluorescence-based labeling of probes is most commonly used to detect binding events, due primarily to simplicity and sensitivity. Examples are amine-reactive dyes such as cyanines (Cy3 and Cy5) or labeling probes with biotin followed by detection with an avidin-fluorescent dye conjugate. CCD cameras or fluorescent slide scanners are used for detection of the labeled probes within defined grid areas. Sandwich immunoassay methods provide increased sensitivity and at the same time reduce non-specific binding, but their use is limited by the availability of paired antibodies. Using a rolling circle amplification (RCA) method can dramatically increase fluorescent signal intensity [70], thus increasing sensitivity in protein microarray detection methods. In this scheme, proteins are labeled with biotin and recognized by an antibody conjugated with a primer to which a circular DNA is hybridized. Fluorescently labeled oligonucleotides are used for elongation of DNA and detection. A universal RCA signal amplification scheme was used [71] for measuring cytokines in a multiplexed format. A total of 75 cytokines were simultaneously measured on an antibody microarray, demonstrating that even larger number of analytes can be measured by this technology. Thus, RCA is a powerful tool for improving the detection limits of protein microarrays by signal amplification.

Current fluorescence-based detection in protein microarrays is limited to two to three independent probes because broad emission profiles exhibited by the dyes used for detection often overlap with each other and with the background fluorescence emitted by substrates, such as the commonly used nitrocellulose. Also, multiple excitation and emission channels are required to detect combinations of fluorescent markers. Quantum dot (QDs) nanoparticles are one alternative to fluorescent dyes. The QDs are semiconducting fluorophores that are extremely bright, resist photobleaching, and have multiplexing capability. Each contains an inorganic fluorescent core, commonly CdSe, coated with a shell of another semiconductor such as CdS or ZnS. The most important property of QDs with reference to protein microarray detection is that they absorb light at broad wavelengths, from UV to visible range and emit light at a very narrow bandwidth, in contrast to fluorescent dyes. Additionally, the QD surfaces may be modified with suitable chemistries for immobilizing biomolecules. Streptavidin-coated QDs in a protein microarray format were used to detect six different cytokines at picomolar concentrations [72], currently the practical limit for multiplexing with QDs. Additional multiplexing strategies have been previously described in detail [23].

Bead-based microarrays offer one solution to multiplexing. Most commercially available methods were developed by Luminex (xMAP platform). The xMAP technology is based on polymer beads incorporated with fluorescent dyes that produce unique signatures. These beads can be conjugated to different molecules to perform multiplexed assays. Up to 100 discrete interactions can be monitored simultaneously using this technology [73], perhaps extended to 500 bead sets in the near future. Antigens or peptides have been attached to the Luminex beads and antibody responses to papillomavirus [74], Epstein-Barr virus [75,76], and *Mycobacterium tuberculosis* [77] were reported. Becton Dickinson (Franklin Lakes, NJ, USA) markets a cytometric bead array (CBA) system, which is another multiplexed bead-based assay that includes flow cytometry for cytokine, chemokine, mouse isotyping, and signaling molecule analysis.

## 10. Label-Free Detection

Label-free detection methods are used for real-time monitoring of binding events and to minimize artifacts caused by using labeled probes. SPR imaging technology is widely used in a microarray format for many applications. SPR is label-free, allows real-time monitoring, and has the additional ability to measure kinetics of the molecular interactions (Figure 1). SPR imaging was developed to simultaneously measure SPR dip changes in an array format, recorded with a CCD camera. Grating-coupled (Flexchip, GE/Biacore, Piscataway, NJ, USA) and prism-coupled (SPRi-Plex, Horiba Scientific, Edison, NJ, USA, ProteOn XPR36, Bio-Rad, Hercules, CA, USA) SPRi systems are some examples of commercially available SPRi microarray systems. A model protein-protein interaction system was reported [78], consisting of the E6 protein of human papillomavirus complexing with the proteins E6AP and p53 using a SPRi system. In another example, an array-based spectral SPR system was used for measuring antibody response to mumps virus infection [79]. In this system the reflected light from the array was collected into a fiber optic spectrometer for analysis. Our laboratory routinely uses SPRi microarrays to validate interactions that were detected during primary microarray screens from a fluorescent probe read-out. Because spot sizes must be large enough for resolution by the commonly used CCD imaging, practical array densities are currently less than 1000 independent immobilized probes.

Additional label-free detection methods have been described. Surface-enhanced laser desorption/ionization time of flight mass spectrometry (SELDI-TOF-MS) has been used as a low throughput method for on-chip purification of proteins, ionization, and detection [80]. However, this approach was not very successful in identifying biomarkers in parasitic diseases, and most markers found in the study were intact host proteins [81,82]. Matrix-assisted laser desorption/ionization (MALDI)-MS in combination with 3-D gel surfaces [83] and silicon nanoporous nanovials [6] were also used for biomarker discovery. The MS-based detection systems are important for specific applications, but there have been only limited advancements in the development of microarray formats, currently limiting use as a primary screening tool. Nanowire-based systems detect changes in conductivity as a result of molecular binding [84,85]. It is possible to make an array of nanowires such that each nanowire is coated with a specific capture molecule. Gantelius *et al.* [86] reported a magnetic bead-based multiplexing format for the detection of auto-antibodies to 12 antigens, comparing the assay results to fluorescence-based detection system. Although the magnetic bead-based system was rapid, it was not sufficiently sensitive for routine use.

## 11. Data Analysis

Despite the rapid progress in protein microarray development standardized methods for data handling and analysis are not universally accepted. Fluorescence scanners made by different commercial vendors use stand-alone software to analyze data, while assay protocols used by investigators may vary widely. Hence, it is often difficult to compare studies reported by different groups, and there is a need to formulate standardized methods for protein microarray data handling and analysis. A number of reports have outlined ways to normalize protein microarray data, mainly for different approaches to antibody microarrays. These normalization methods include concentration-dependant analysis [87,88], spiked internal standards [89], and algorithm-based [90], and all have significant pros and cons. Invitrogen/Life Technologies (Carlsbad, CA, USA) developed a data analysis package (ProtoArray Prospector) specifically designed for protein antigen microarrays, including a suite of statistical methods. BioArray software environment (BASE), a software package for microarray data management and analysis (http://base.thep.lu.se) developed by Lund University, Sweden, also addresses some of the issues we have outlined. However, it remains to be seen how rapidly standards will be adopted by the research community.

## 12. Conclusions

Antibodies are primary biomarkers of infection that are commonly used for diagnosis, measuring vaccine efficacy, and discovery of disease interventions. Protein microarrays are important tools for measuring these complex antigen-antibody interactions and other host-pathogen interactions at different stages of the disease. The major impediments to development of protein microarrays are the inherent complexity of proteins themselves, availability of well-characterized antibodies to infectious agents, and standardized statistical methods for analysis of data. However, we expect many of these issues to be resolved in the coming years as these tools are used more frequently in infectious disease research.
